# Unilateral Botulinum Neurotoxin-A Injection into the Striatum of C57BL/6 Mice Leads to a Different Motor Behavior Compared with Rats

**DOI:** 10.3390/toxins10070295

**Published:** 2018-07-17

**Authors:** Veronica Antipova, Andreas Wree, Carsten Holzmann, Teresa Mann, Nicola Palomero-Gallagher, Karl Zilles, Oliver Schmitt, Alexander Hawlitschka

**Affiliations:** 1 Institute of Anatomy, Rostock University Medical Center, Gertrudenstrasse 9, D-18057 Rostock, Germany; veronica.antipova@medunigraz.at (V.A.); Teresa.Mann@med.uni-rostock.de (T.M.); oliver.schmitt@med.uni-rostock.de (O.S.); alexander.hawlitschka@med.uni-rostock.de (A.H.); 2 Gottfried Schatz Research Center for Cell Signaling, Metabolism and Aging, Macroscopic and Clinical Anatomy, Medical University of Graz, Harrachgasse 21/1, A-8010 Graz, Austria; 3 Institute of Medical Genetics, Rostock University Medical Center, Ernst-Heydemann-Strasse 8, D-18057 Rostock, Germany; carsten.holzmann@med.uni-rostock.de; 4 Institute of Neuroscience and Medicine INM-1, Research Center Jülich, D-52425 Jülich, Germany; n.palomero-gallagher@fz-juelich.de (N.P.-G.); k.zilles@fz-juelich.de (K.Z.); 5 Department of Psychiatry, Psychotherapy and Psychosomatics, Medical Faculty, RWTH Aachen, D-52062 Aachen, Germany; 6 JARA—Translational Brain Medicine, D-52062 Aachen, Germany

**Keywords:** botulinum neurotoxin-A, basal ganglia, interspecies differences in motor behavior, mouse, rat, interneurons

## Abstract

Different morphological changes in the caudate-putamen (CPu) of naïve rats and mice were observed after intrastriatal botulinum neurotoxin-A (BoNT-A) injection. For this purpose we here studied various motor behaviors in mice (*n* = 46) longitudinally up to 9 months after intrastriatal BoNT-A administration as previously reported for rats, and compared both outcomes. Apomorphine- and amphetamine-induced rotational behavior, spontaneous motor behavior, as well as lateralized neglect were studied in mice after the injection of single doses of BoNT-A into the right CPu, comparing them with sham-injected animals. Unilateral intrastriatal injection of BoNT-A in mice induced significantly increased contralateral apomorphine-induced rotations for 1 to 3 months, as well as significantly increased contralateral amphetamine-induced rotations 1 to 9 months after injection. In rats (*n* = 28), unilateral BoNT-A injection also induced significantly increased contralateral apomorphine-induced rotations 3 months after injection, but did not provoke amphetamine-induced rotations at all. Lateralized sensorimotor integration, forelimb preference, and forelimb stepping were significantly impaired on the left side. The differences in motor behaviors between rats and mice may be caused by different BoNT-A effects on cholinergic and catecholaminergic fibers in rat and mouse striata, interspecies differences in striatal receptor densities, and different connectomes of the basal ganglia.

## 1. Introduction

Parkinson’s disease (PD) is a common chronic progressive age-related neurodegenerative movement disorder characterized by the loss of dopaminergic neurons in the substantia nigra pars compacta and, subsequently, of dopamine in the caudate-putamen (CPu) [1,2,3]. Dopamine deficit causes a profound impairment of neuronal circuits in the basal ganglia [4,5,6,7], and particularly an increased release of acetylcholine by tonically active striatal interneurons [8,9,10,11,12]. Anticholinergic drugs are used to antagonize striatal hypercholinism in PD, but this treatment often elicits adverse side effects [13,14,15]. In order to circumvent general anticholinergic drug effects, we tested a local intrastriatal injection of botulinum neurotoxin-A (BoNT-A) [16,17,18,19,20].

Indeed, in the experimental 6-OHDA-induced hemiparkinsonian (hemi-PD) rat model, intrastriatal application of BoNT-A abolished apomorphine-induced rotational behavior—most probably by blocking acetylcholine, the release of cholinergic terminals, and/or inducing changes in receptor densities [16,17,18,19,20,21,22,23]. Moreover, the unilateral intrastriatal BoNT-A injection induced rotational behavior in naïve rats without 6-OHDA-induced hemi-PD. Following the injection of 1 ng BoNT-A into the right CPu, rats showed 2–4 apomorphine-induced rotations per min in the direction of the injection site for two months, and rotated tentatively to the contralateral side thereafter [19].

To date, behavioral effects of intrastriatal BoNT-A injections were studied in rats, but not in mice. This would be interesting, as comparative morphological studies of the striata of BoNT-A-injected rats and mice striata showed obvious differences in the structural changes of choline acetyltransferase-immunoreactive (ChAT-ir) and tyrosine hydroxylase-immunoreactive (TH-ir) fibers [19,20,24], in addition to similarities concerning unchanged CPu volume and the number of cholinergic interneurons. Rats and mice differed in the induction of BoNT-A-induced varicosities: both kinds of these varicosities were found in rats [16,17,19,20,21], whereas only ChAT-ir BoNT-A-induced varicosities were observed in mice [24].

As naïve mice and rats reacted morphologically in a different manner following intrastriatal BoNT-A injection, we sought to determine whether these species differences would also hold true for motor behavior. Thus, mouse motor behavior was scored after unilateral BoNT-A injection into the right CPu using apomorphine- and amphetamine-induced rotations. Spontaneous behavior was tested using the cylinder and stepping tests, lateralized sensorimotor activity by the corridor task, and cerebellar ataxia by hindlimb clasping. To evaluate temporally restricted effects, mice were tested up to 9 months after BoNT-A injection. A potential dose-dependency was evaluated by using dosages of 25 and 50 pg BoNT-A.

It is known that mice are very sensitive to botulinum toxins and also differ in many respects from rats [25,26,27,28,29,30,31,32,33]. However, there are currently no studies evaluating the behavioral outcome of intrastriatally applied BoNT-A in naïve mice. Moreover, the effect of BoNT-A in naïve mice must be understood prior to evaluation of intrastriatal BoNT-A application in mice models of hemi-PD and genetic PD. The BoNT-A dosage used for intrastriatal injection for the treatment of experimental hemi-PD in rats was based on the LD50 and recent intracerebral BoNT-A injections [34,35,36,37,38]. In Wistar rats we found a well-tolerated and effective dosage at 1 ng BoNT-A per CPu, whereas 5 ng were mortal [19]. In the C57BL/6 mice, dosages of 25–50 pg BoNT-A per striatum were found to be appropriate [24].

Studying the behavioral effect of unilateral intrastriatal BoNT-A application in naïve mice also seemed to be important, since the effect of therapeutic BoNT-A applications is explored in increasingly studied genetic Parkinsonian mouse models [39,40,41,42,43,44,45].

## 2. Results

### 2.1. Body Weight

**Figure 1:**
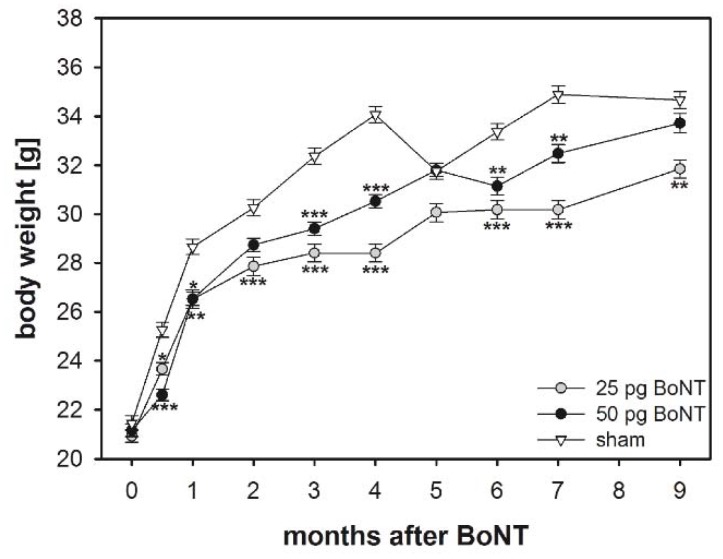
Body weight over time. Asterisks indicate significant differences (25 pg, *n* = 15; 50 pg, *n* = 20) compared to the sham group (*n* = 11) (* *p* < 0.05, ** *p* < 0.01, *** *p* < 0.001). Data are means ± SEM.

Changes in body weight were evaluated as an index of general adverse effects of BoNT-A application. The mice weighed 18–24 g at the time of BoNT-A or vehicle injection. Thereafter, body weight increased in all experimental groups (Figure 1). Increase in body weight over time was significantly (F_2, 50_ = 15.5999, *p* < 0.001) lower in mice injected with BoNT-A than in those receiving vehicle substance. However, 9 months after BoNT-A injection, the 25 pg BoNT-A mice weighed 31.85 ± 0.37 g (mean ± SEM), the 50 pg BoNT-A mice 33.72 ± 0.40 g, and thus did not differ from the sham group, which weighed 34.66 ± 0.35 g (Figure 1).

### 2.2. Apomorphine-Induced Rotations

#### 2.2.1. Mice

**Figure 2:**
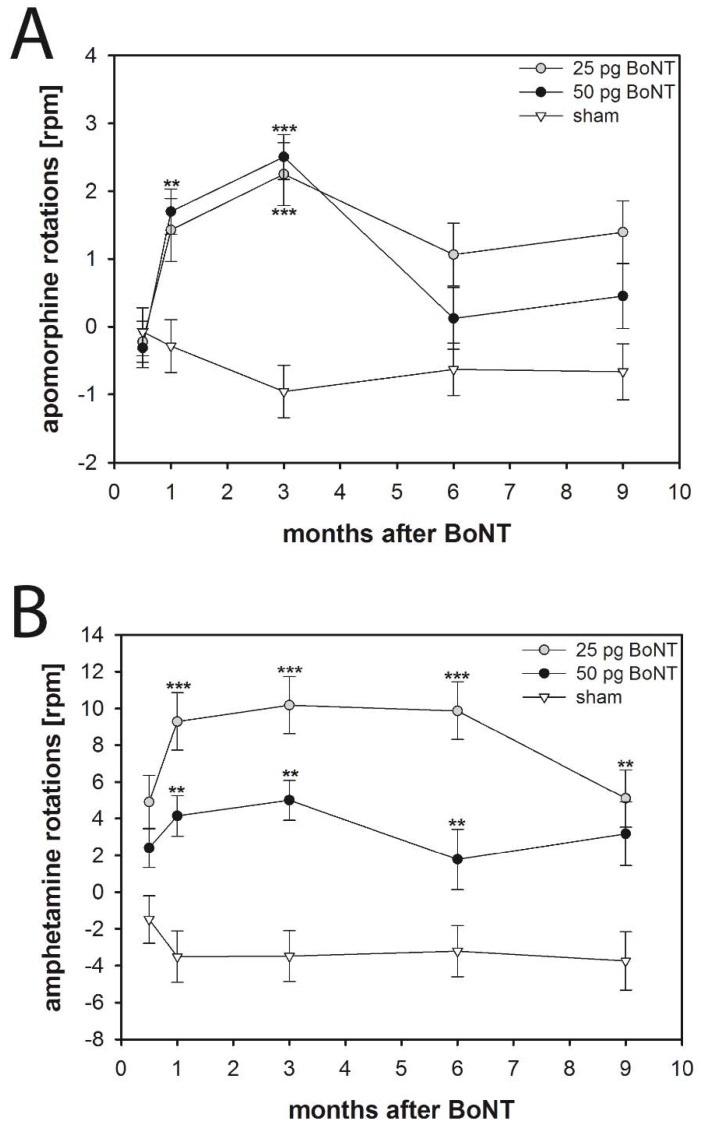
(**A**) Apomorphine- and (**B**) amphetamine-induced rotations in mice treated with intrastriatal BoNT-A (25 pg, *n* = 15; 50 pg, *n* = 20) or vehicle (*n* = 11). (**A**) BoNT-A in either dosage caused a significant increase of the apomorphine-induced turning rate 1 and 3 months after injection. (**B**) BoNT-A in both dosages resulted in significantly increased amphetamine-induced rotations 1–9 months after injection, while sham injections did not change rotational behavior. Asterisks indicate significant differences compared with the sham group (** *p* < 0.01, *** *p* < 0.001). Data are means ± SEM.

In order to test the effect of a unilateral intrastriatal BoNT-A injection on drug-induced motor behavior we used apomorphine- and amphetamine-induced rotation tests. Half a month after injection of 25 or 50 pg BoNT-A or vehicle into the right CPu mice showed no apomorphine-induced rotation behavior (Figure 2A). Vehicle application did not induce rotational behavior in the subsequent 9 months. However, both applications of 25 and 50 pg BoNT-A resulted in significant (F_2, 50_ = 6.150, *p* = 0.004) anti-clockwise, i.e., contralateral, apomorphine-induced rotations with net rotations of about 2 per minute after a post-injection survival of 1 and 3 months (Figure 2A). Six and 9 months after BoNT-A, rotational behavior decreased to values similar to those observed in the vehicle-treated group.

#### 2.2.2. Rats

**Figure 3:**
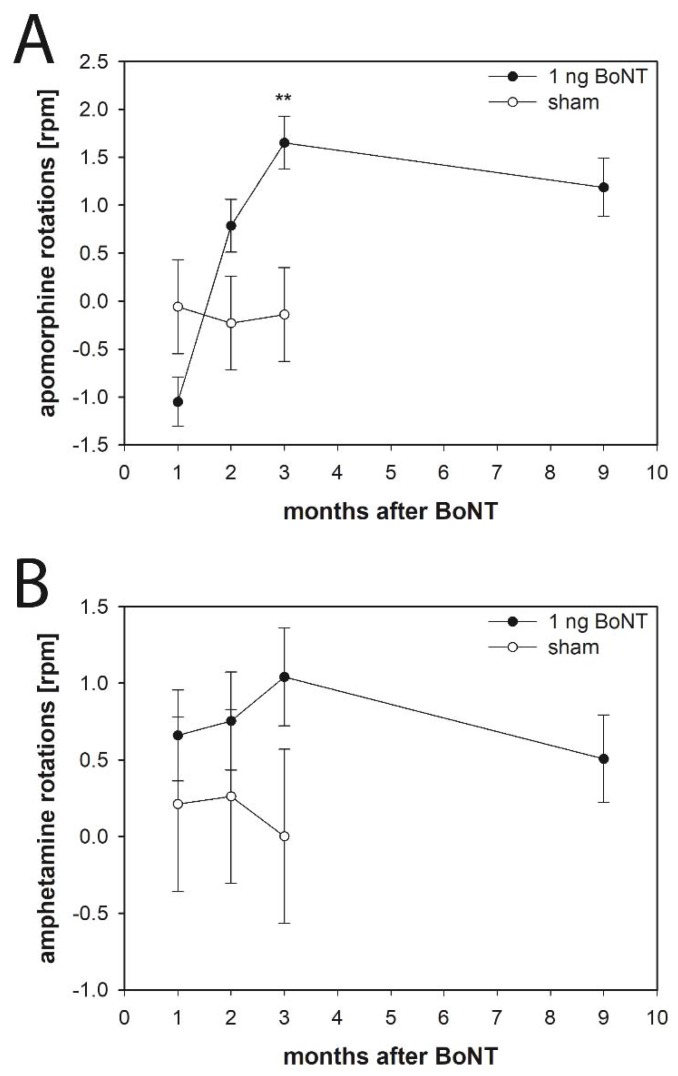
(**A**) Apomorphine- and (**B**) amphetamine-induced rotations in rats intrastriatally injected with BoNT-A (*n* = 22) or vehicle (*n* = 6). (**A**) 1 ng BoNT-A caused a significant anti-clockwise apomorphine-induced turning 3 months after injection, (**B**) but did not influence amphetamine-induced rotation behavior significantly 1–9 months after BoNT-A as compared to the sham group. Asterisks indicate significant differences compared with the sham group (** *p* < 0.01). Data are means ± SEM.

Following apomorphine injection rats significantly rotated contralaterally to the intrastriatal BoNT-A application with rotations of approximately 1.5 to 2 per minute 3 months after 1 ng BoNT-A (Figure 3A).

### 2.3. Amphetamine-Induced Rotations

#### 2.3.1. Mice

After intrastriatal injection of 25 or 50 pg BoNT-A, the mice showed significantly increased (F_2, 48_ = 15.360, *p* < 0.001) amphetamine-induced rotations compared to the vehicle group from 1 to 9 months post injection (Figure 2B). Whereas application of BoNT-A was associated with strong amphetamine-induced anti-clockwise net rotations of approximately 4–10 per min, vehicle injection resulted in clockwise rotations of approximately 3 per min (Figure 2B).

#### 2.3.2. Rats

In rats, amphetamine application revealed no significant effect of unilateral intrastriatal BoNT-A as compared with vehicle injection (Figure 3B).

### 2.4. Cylinder Test

**Figure 4:**
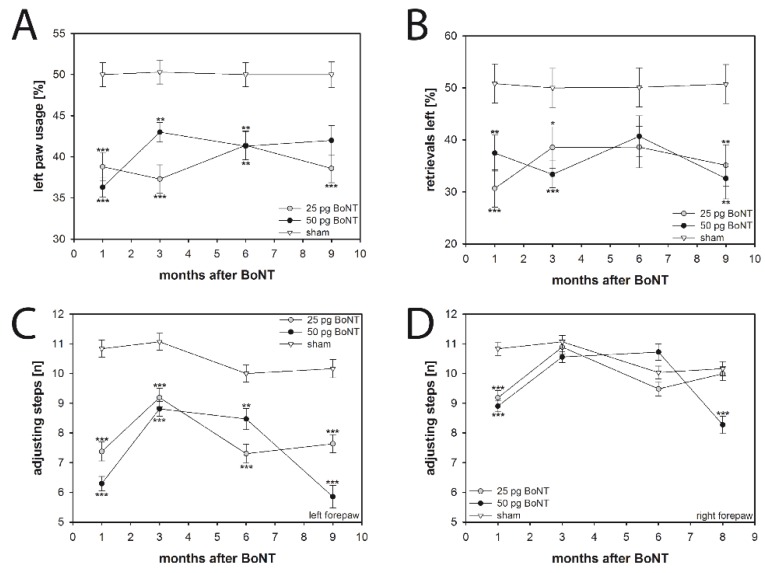
(**A**) The cylinder test of BoNT-A-injected mice (25 pg, *n* = 15; 50 pg, *n* = 20) of both dosages revealed a significantly lower use of the left forepaw at all time points compared with sham-injected mice (*n* = 11). (**B**) In the corridor task, BoNT-A-injected mice of both dosages (25 pg, *n* = 11; 50 pg, *n* = 12) retrieved pellets significantly less often from the left side compared to the sham group (*n* = 10). (**C**,**D**) The stepping test revealed constant adjusting steps of sham-treated mice (*n* = 10). (**C**) Right side BoNT-A-injected mice of both dosages (25 pg, *n* = 11; 50 pg, *n* = 10) displayed significantly decreased left forepaw adjusting steps 1–9 months after surgery, whereas (**D**) the number of adjusting steps of the right forepaw only differed significantly from the sham group one month after BoNT-A injection. However, the 50 pg BoNT-A group also showed significantly fewer right forepaw adjusting steps 9 months after injection. Asterisks indicate significant differences compared with the sham group (* *p* < 0.05, ** *p* < 0.01, *** *p* < 0.001). Data are means ± SEM.

The cylinder test evaluates asymmetries in spontaneous forelimb use during exploratory activity in a novel environment. Sham injection did not induce any laterality of forepaw usage during the whole testing period up to 9 months; left and right forepaws were used equally often in the sham group (Figure 4A). In contrast, BoNT-A-injected mice, irrespective of the dosage and survival time, exhibited a significantly reduced (F_2, 52_ = 31.971, *p* < 0.001) use of the left forepaw of about 40% (Figure 4A).

### 2.5. Corridor Task

Lateralized sensorimotor integration was assessed using the corridor test, which depends on the rodent’s ability to retrieve food from either side of its body. Animals of the vehicle group made an equivalent number of retrievals (about 50% of total retrievals from both the left and right sides of the corridor) over the whole testing period (Figure 4B). However, mice of both BoNT-A groups showed a significant neglect (F_2, 40_ = 11.506, *p* < 0.001) of the corridor side contralateral to the BoNT-A injection side. These mice retrieved only about 35% of the sugar pellets from the left corridor side during the whole observation period (Figure 4B).

### 2.6. Stepping Test

The motor activity of the forelimbs was estimated using a mouse-friendly version of the stepping test. Adjusting forepaw steps were measured on the injected (right) and noninjected (left) sides. Mice of the sham group made approximately 10–11 steps with either forepaw during the whole testing time (Figure 4C,D). Mice of both BoNT-A groups exhibited significantly fewer adjusting steps (F_2, 42_ = 99.773, *p* < 0.001) with their left forepaws up to 9 months after BoNT-A (Figure 4C). A reduction in adjusting steps with the right forepaw was seen in BoNT-A-injected mice of both dosages 1 month after BoNT-A (Figure 4D).

### 2.7. Hindlimb Clasping

We evaluated neurological abnormalities using the hindlimb clasping test. Mice of the two BoNT-A groups and the sham group did not show any pathological hindlimb clasping during the whole testing period from 1 to 9 months; the hindlimbs of all mice were consistently splayed outward and away from the abdomen resulting in an assigned score of 0.

## 3. Discussion

Previous studies of unilateral injections of BoNT-A into the CPu of male C57BL/6 mice showed that the number of ChAT-ir interneurons was unaltered in the CPu, and ChAT-ir BoNT-A-induced varicosities were visible. Both results are comparable with findings in the rat [16,17,18,19,20]. However, in contrast to rats, there were no TH-ir BoNT-A-induced varicosities in BoNT-A-injected mouse. As BoNT-A application seemed to have a different influence on cholinergic and dopaminergic axons in the mouse striatum [24], the CPu-associated motor behavior was studied. Moreover, a possible temporally occurring BoNT-A effect as seen in previous studies in rats [16,17,18,19,20,21] was evaluated by testing mice up to 9 months after BoNT-A injection. Dose dependency was studied by using dosages of 25 and 50 pg BoNT-A. As a significant dose dependency within the evaluated parameters was not obvious, the results of mice receiving dosages of 25 and 50 pg BoNT-A will be discussed together.

We found that unilateral intrastriatal BoNT-A injection in naïve mice led to significant contralateral amphetamine- and apomorphine-induced rotations as well as to significant impairments of the left (i.e., contralateral) side with respect to lateralized sensorimotor integration, forelimb usage, and forelimb stepping.

### 3.1. Basal Ganglia Circuitry after BoNT-A Injection

Locally injected BoNT-A is thought to act in two main ways in the striatum, where it blocks the acetylcholine release of the tonically active cholinergic interneurons [46,47,48], counteracts the D_2_ receptor upregulation found in hemi-PD rats, and reduces D_2_ receptor concentrations in naïve rats [22,23]. The assumed BoNT-A-induced reduction of acetylcholine concentration in the injected CPu reduces the firing activity of medium spiny neurons projecting to (i) the external globus pallidus, i.e., those which are part of the indirect basal ganglia loop, and (ii) to the internal globus pallidus, i.e., those which are part of the direct basal ganglia loop [49,50,51,52,53].

The effects of BoNT-A on the reduction of cholinergic transmission and of D_2_ receptor concentrations seemingly underlie movement initiation deficits, akinesia and reduced spontaneous use of the contralateral forelimbs [54,55,56,57].

### 3.2. Body Weight

Body weight increased in all three experimental groups from two weeks up to 9 months after intrastriatal BoNT-A or vehicle injection. However, mice injected with either of the BoNT-A dosages weighed less compared to the sham group two weeks after injection onwards, although the differences diminished after 9 months. These differences might point at a mild temporal toxicity of BoNT-A after the stereotactic injection.

### 3.3. Spontaneous Motor Tests

#### 3.3.1. Spontaneous Forelimb Use

The cylinder test evaluates locomotor asymmetry and forelimb use in rodent models of central nervous system disorders by assessing the innate drive to explore a novel environment by rearing and leaning their forepaw against the wall of the glass cylinder [58]. Unilaterally sham-injected mice used the left and right paws symmetrically, i.e., about 50% each. Right side intrastriatal application of BoNT-A significantly reduced the use of the left paw.

Seemingly, the impairment of motor initiation deficit for voluntary movements of the contralateral forelimb is a specific result of BoNT-A injection into the CPu since it decreases firing of GABAergic medium spiny neurons to the internal globus pallidus in the direct loop, and therefore inhibits the ventrolateral thalamic nucleus. On the other hand D_2_ receptor-bearing medium spiny neurons increase inhibition of the external globus pallidus in the indirect loop, and resulted in a more actively firing internal globus pallidus via reduced inhibition of the spontaneously active subthalamic nucleus. Therefore, the inhibited neurons of the ventrolateral thalamic nucleus are not able to sufficiently activate the premotor cortex via both loops.

#### 3.3.2. Sensorimotor Integration

The corridor task, originally designed to study unilateral sensorimotor integration impairments in rats [59,60], was adapted for experiments in mice [61]. Mice injected with 25 or 50 pg BoNT-A into the right striatum retrieved pellets significantly less often from the left side during the testing period up to 9 months after BoNT-A, whereas sham-injected mice behaved symmetrically. As is the case for the forepaw preference studied in the cylinder test, the motor initiation deficit for voluntary movements of the contralateral forelimb is probably a specific result of the BoNT-A injection into the CPu. Both the reduction of striatal cholinergic transmission and the reduction of D_2_ receptor density causes changes in the basal ganglia circuitry resulting in a reduced initiation of movements of the contralateral body via crossed motor efferents [57,62,63].

#### 3.3.3. Forelimb Adjusting Steps

In our study, we used the mouse-friendly version of the stepping test described by Blume et al. [64] and modified by Heuer et al. [65]. Right sided intrastriatal application of 25 and 50 pg BoNT-A clearly reduced the stepping frequency of the left forepaw up to 9 months after injection compared to sham group. A reduced initiation of movements of the paw contralateral to the BoNT-A application via crossed motor efferents is reasonable for the outcome of this behavioral test [66,67,68]. Interestingly, the intrastriatal BoNT-A injection into the right CPu also results in a short-term reduction of right paw adjusting steps. One month after vehicle injection mice made 10.8 ± 0.2 steps; at the same time the 25 pg BoNT-A group made 9.2 ± 0.2, and the 50 pg BoNT-A group 8.9 ± 0.2 steps. This phenomenon is not fully understood, as the right forepaw steps should be unaltered. The mouse-friendly design of the stepping test has also been applied in the mouse MTPT model [64] resulting in bilateral impairment, and also following different right side models of dopaminergic lesioning to induce unilateral parkinsonian-like symptoms [65,69,70]. Unilateral dopaminergic depletion, however, led to contradicting results in the same test. Heuer et al. [65] stated that the number of steps is reduced in both paws to about 80% compared to sham-injected mice irrespective of the experimental approach inducing unilateral or bilateral striatal dopamine reduction. In contrast, Glajch et al. [69] reported a ratio of contralateral-to-ipsilateral steps of about 0.2 after unilateral 6-OHDA lesion of the medial forebrain bundle. Boix et al. [70] reported a ratio of approximately 6 to 27%, depending on the 6-OHDA dosage used. Thus, both groups show a clear unilateral effect. However, neither Glajch et al. [69] nor Boix et al. [70] mention the absolute number of steps made by the ipsilateral paw, which would be important for comparison with our results.

#### 3.3.4. Hindlimb Clasping

Hindlimb clasping has been shown to occur in various neurodegenerative mouse models [71,72] including PD models [73,74]. All our mice, irrespective of BoNT-A or vehicle injection, never showed pathological hindlimb clasping during the whole testing period from 1 to 9 months. We interpret the absence of this pathological behavior in our model as indirect evidence that the BoNT-A dosages used are not generally toxic in the striatum.

### 3.4. Drug-Induced Rotation Tests

#### 3.4.1. Apomorphine-Induced Rotations

There is an apomorphine-induced rotational behavior of BoNT-A-treated mice and rats. Mice with 25 and 50 pg BoNT-A injected intrastriatally showed significant contralateral apomorphine-induced rotations after a survival of 1 and 3 months with rotations of about 2 per minute. Thereafter, rotational behavior decreased to values not significantly different from those of the vehicle group. These measurements corroborate data obtained in rats (Figure 3A), which also showed a significantly altered behavior with rotations of approximately 1.5 to 2 per minute 3 months after intrastriatal injection of 1 ng BoNT-A.

Apomorphine-induced rotations are mainly due to binding of the drug to D_2_ receptors that are distributed unequally in both striata. Following apomorphine application, hemi-PD rats and hemi-PD mice rotate to the body side contralateral to the dopamine-depleted hemisphere, which has a higher D_2_ receptor concentration than the contralateral one [65,70,75]. Acetylcholine is reduced in BoNT-A-injected striata, and thus, the activation of all medium spiny neurons is reduced. Moreover, striatal receptor concentration measurements investigated in BoNT-A-treated hemi-PD rats speak in favor of a BoNT-A-induced reduction of D_2_ receptors in the respective CPu [22,23].

Considering that the majority of striatal D_2_ receptors are located on the D_2_ receptor bearing medium spiny neuron, an unaltered dopamine concentration in the CPu would result in a movement deficit of the contralateral body side via the indirect basal ganglia loop in BoNT-A-treated striata. However, if the majority of striatal D_2_ receptors are located on the presynaptic terminals of the dopamine afferents from the substantia nigra pars compacta, then dopamine release should be increased due to reduced inhibition by D_2_ autoreceptors on dopamine terminals [76]. The hypothetically increased striatal dopamine concentration in the BoNT-A-injected CPu would result in an increased movement of the contralateral body side via the indirect basal ganglia loop as suggested by Da Cunha et al. [77]. As all tests for spontaneous motor behavior after striatal BoNT-A revealed an initiation deficit in the contralateral forelimb, the functional significance of D_2_ autoreceptors for these non-drug-induced behaviors seemed limited.

Since apomorphine is mainly a D_2_ receptor agonist, its application possibly reverses the alterations in basal ganglia circuitry induced by BoNT-A by shifting the dopamine-mediated functional significance from D_2_ receptor-bearing medium spiny neuron to D_2_ autoreceptor-bearing dopamine terminals, thus resulting in a stronger movement initiation in the contralateral body musculature. If dopamine release by the disinhibited dopamine terminals exceeds the apomorphine-induced disinhibition of the D_2_ receptor-bearing medium spiny neuron, then we can assume the occurrence of a mildly increased contralateral forelimb activity via deactivation of the medium spiny neuron of the indirect basal ganglia loop, which would result in the contralateral rotation behavior [66,67].

#### 3.4.2. Amphetamine-Induced Rotations

Mice with intrastriatal injection of 25 or 50 pg BoNT-A show clear anti-clockwise rotations of about 4–10 per min following the application of amphetamine (Figure 2B). In contrast, comparable experiments in rats revealed no significant effect of BoNT-A injections as compared with vehicle injection (Figure 3B). The obvious difference in BoNT-A-inducible amphetamine rotations between mice and rats is not fully understood.

Amphetamine mainly increases the extracellular dopamine concentration by different mechanisms: it competitively inhibits dopamine uptake via dopamine transporter, facilitates the movement of dopamine from the vesicle into the cytoplasm, and promotes DAT-mediated reverse transport of dopamine into the synaptic cleft independent of action potential-induced vesicular release [78]. Additionally, microdialysis studies in rats revealed amphetamine-induced increased extracellular concentrations of glutamate, aspartate, GABA, taurine, glycine, serotonin, acetylcholine, and of different peptides [79,80,81,82,83,84,85,86].

There is a notable species difference regarding amphetamine-induced contralateral rotation behavior in unilaterally BoNT-A-injected animals, since it is present in mice, but not in rats. Three aspects will be discussed: (1) different effects on axon terminals after intrastriatal BoNT-A injections in mice and rats, (2) the concentrations of the most frequent transmitter receptors in the CPu of naïve mice and rats, and (3) the connectome of the basal ganglia of mice and rats.

Hawlitschka et al. [24] showed that striata from mice and rats react differently after BoNT-A injection with respect to the appearance of TH-ir BoNT-A -induced varicosities [24], since they are consistently found in rats, but were never seen in mice [19,24]. BoNT-A-induced varicosities can be interpreted as a sign of structural alterations induced by BoNT-A. The difference in the occurrence of BoNT-A-induced varicosities between mice and rats might be based on the synaptic vesicle glycoprotein C (SV2C) receptor responsible for the internalization of BoNT-A into the neuron [87,88]. Unfortunately, no data exist concerning differences of SV2C affinity or susceptibility between the CPu of rats and mice [87,88,89,90,91,92,93,94,95]. It cannot be ruled out that the dopaminergic fibers are differently influenced by BoNT-A and thus the balance or interaction of various transmitter systems on the functional outcome underlying the amphetamine-induced rotation behavior of intrastriatally applied BoNT-A may differ [79,96,97,98,99,100,101]. In contrast to the interspecies differences in BoNT-A-induced varicosities, ChAT-ir BoNT-A-induced varicosities were consistently found in CPu of both rats and mice following intrastriatal BoNT-A.

#### 3.4.3. Receptors and Connectomics of the CPu

To analyze possible interspecies differences of transmitter systems in the CPu of control rats and mice, we compared the multireceptor fingerprints (Figure S1) of Wistar rats (*n* = 6, data from [23,102,103]) and of C57Bl/6 mice (*n* = 6; data from [40]). Additionally, the connectome of the mouse basal ganglia generated in a high throughput tract tracing study published as Alan Atlas connectome [104] is compared with rat data (Figure S2). Details to multireceptor fingerprints and connectomes are provided in the Supplement.

## 4. Conclusions and Future Perspectives

BoNT-A unilaterally injected into the CPu in naïve mice differentially affected various motor behaviors. Lateralized sensorimotor integration, forelimb preference, and forelimb stepping were significantly impaired contralateral to the injected side. Unilateral intrastriatal BoNT-A induced significant contralateral amphetamine-induced rotations from 1 to 9 months post injection, which is completely opposite to the reaction found in rats. The differences in motor behavior induced by unilateral intrastriatal BoNT-A injections between rats and mice is possibly caused by different effects of BoNT-A on TH-ir fibers in rat and mouse striata, interspecies differences in striatal receptor densities, and different connectomes of the basal ganglia between mice and rats.

Local injection of BoNT-A into the striatum of hemi-PD rats is thought to decrease the local release of acetylcholine therein. Hypercholinism of the striatum caused by acetylcholine released from disinhibited tonically active cholinergic interneurons is held responsible for a disturbed basal ganglia circuitry and, consequently, for motor and behavioral dysfunctions [9,46,48,105]. One possible approach to treat PD is the reduction of the hypercholinism using oral anticholinergic drugs [106,107]. Due to the systemic drug application, adverse side effects such as anticholinergic syndrome and dyskinesia are common [13,14,15,108]. In the experimental hemi-PD rats, BoNT-A injected locally into the CPu is beneficial for 3 to 6 months with respect to apomorphine-induced rotational behavior [16,17,18,19,20,21]. Future experiments will cover two topics: First, repeated intrastriatal BoNT-A injections in hemi-PD rats every 6 months should test whether motor behavior could be improved for a longer time period, which would be a prerequisite for the clinical application of BoNT-A [109,110,111,112]. Second, extending our studies of intrastriatal BoNT-A injections to naïve mice, BoNT-A will be injected into mice of various parkinsonian models including those with alterations of relevant PD-associated genes to obtain further insights into PD-related etiology [39,40,113,114,115].

In conclusion, locally applied BoNT-A, or other botulinum neurotoxins, could be useful in treating brain dysfunctions requiring a deactivation of local brain activity [116]. Advantageously, the effect of local BoNT-A is time-limited and reversible. It can be speculated that following prospective experiments in primates botulinum neurotoxins might be applied as an effective and individually-tailored “chemical neurosurgical approach” [116,117].

## 5. Materials and Methods

### 5.1. Animals

A total of 46 young adult male C57BL/6 mice (Charles River Wiga, Sulzfeld, Germany) weighing 18–24 g and 28 Wistar rats (strain Crl: WI BR, Charles River Wiga, Sulzfeld, Germany) with a body weight of 280–320 g were used in this study. Animals were housed in standard cages in a temperature-controlled room (22 ± 2 °C) under 12 h light/12 h dark conditions with free access to food and water. All procedures were approved by the State Animal Research Committee of Mecklenburg-Western Pomerania (LALLF M-V/TSD/7221.3-1.1-053/08, LALLF M-V/TSD/7221.3-1.1-003/13 from 26 April 2013 and 13 April 2016).

### 5.2. BoNT-A application

**Figure 5:**
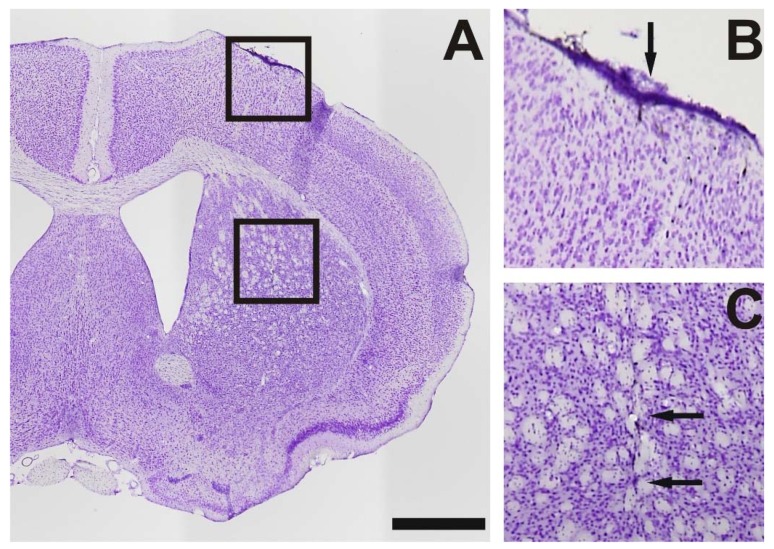
(**A**) A coronal Nissl-stained brain section 30 µm thick of a mouse treated intrastriatally with 25 pg BoNT-A 6 months before sacrifice. (**B**,**C**) Higher magnifications of the boxes in (**A**). In (**B**) the needle tract through the cortex is marked by an arrow, in (**C**) the injection channel in the striatum is indicated by two arrows. Scale bar applies to (**A**): 1 mm.

In the mice, surgery was conducted under aseptic conditions and animals were deeply anesthetized with ketamine (75 mg/kg, bela-pharm Vechta, Germany)/xylazine (5.8 mg/kg, Rompun^®^, Bayer, Germany), mounted in a mouse adapter (Stoelting, Wood Dale, IL, USA), and fixed in a rat stereotactic apparatus (Kopf, Tujunga, CA, USA). The skull was opened with a dental drill and the mice received an injection of 1 µL BoNT-A solution (Lot No. 13028A1A; List, Campbell, CA, USA, purchased via Quadratech Diagnostics, Surrey, UK) containing a total of 25 pg or 50 pg BoNT-A dissolved in PBS + 0.1% BSA into the right CPu delivered over 4 min using a 26-gauge 5 µL Hamilton syringe, at a rate of 0.25 µL per minute (Figure 5). The sham group received 1 µL BoNT-A vehicle solution. The injection coordinates with reference to bregma were: anterior-posterior = +0.65 mm, lateral = −1.6 mm, and vertical = −3.0 mm from dura, respectively [118] (Figure 5).

Rats were injected either with 1 ng BoNT-A (*n* = 22) or the vehicle solution (sham, *n* = 6) into the right striatum under ketamine (50 mg/kg)/xylazine (4 mg/kg) anesthesia. The BoNT-A solution was injected at two sites, each injection consisting of 0.5 ng BoNT-A solved in 1 µL PBS + 0.1% BSA. Sham-injected rats received the vehicle solution. The coordinates according to bregma were: anterior-posterior = +1.3 mm/−0.4 mm, lateral = −2.6 mm/−3.6 mm and ventral = −5.5 mm/−5.5 mm, respectively [119].

We founded our experiments on the weight values of BoNT-A, i.e., 25 to 50 pg per mouse CPu and 1 ng per rat CPu. In mice the LD_50_ value after intraperitoneal injection is about 0.25–1.15 ng/kg BoNT-A [120]. Thus, in our experiments a dosage of 50 pg BoNT-A per mouse, injected intrastriatally, is below the classical LD_50_, and well tolerated. In clinical use the normal amount of BoNT-A (Xeomin, Dysport, Botox and others) is about 200 to 300 units at maximum per patient per session depending on the clinical symptoms and site of application. Remarkably, the clinical efficacy of a “unit” is reported differently by the various manufactures (Botox: 5 ng correspond to 100 units; Dysport: 4.35 ng correspond to 500 units; Xeomin: 0.6 ng correspond to 100 units) [121]. These uncertainties relay on the nonstandardized LD_50_ bioassays required to achieve the median lethal dose = LD_50_ (depending on mouse strain, sex, age, volume and route of injection) [122].

### 5.3. Body Weight

Body weights were measured before stereotactic surgery and 0.5, 1, 2, 3, 4, 5, 6, 7, and 9 months after injection of BoNT-A or vehicle.

### 5.4. Behavioral Testing

**Figure 6:**
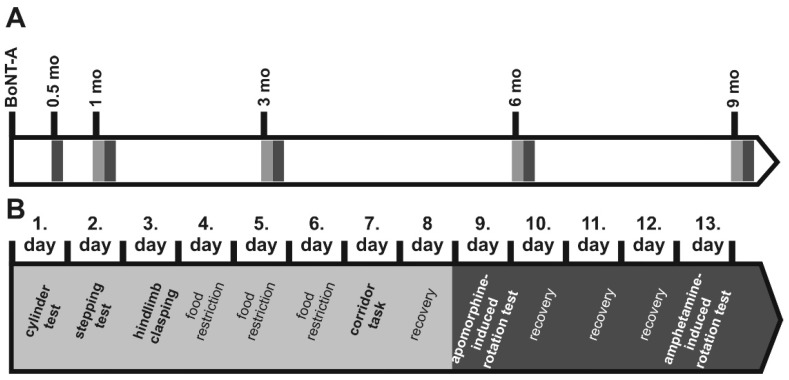
(**A**) Time schedule of the stereotactic application of BoNT-A in mice and behavioral tests. Light grey rectangles symbolize batteries of non-drug-induced behavior tests, dark grey rectangles the subsequently performed apomorphine- and amphetamine-induced rotation tests. (**B**) Detailed visualization of single behavior test batteries. Non-drug-induced tests were performed on the days presented in the light grey part of the time line, drug-induced tests on the days of the dark grey part. The non-drug-induced tests were performed as follows: On the first day the mice underwent the cylinder test until 30 consecutive touches of the glass wall with the forepaws were done. On the second day the stepping test and on the third day the hindlimb clasping were carried out three times per mouse, respectively. On the following 3 days mice underwent food restriction and on the seventh day the corridor task was performed. Following recovery the drug-induced rotation tests were conducted.

Three experimental mouse groups were behaviorally tested: (1) animals receiving 25 pg BoNT-A (25 pg BoNT-A group, *n* = 15), (2) animals receiving 50 pg BoNT-A (50 pg BoNT-A group, *n* = 20), and (3) animals receiving vehicle (sham group, *n* = 11). During the experimental period 5 mice died. Smaller groups were evaluated for the stepping test and the corridor task (25 pg, *n* = 11; 50 pg, *n* = 12; sham, *n* = 10). All mice were adapted to the examination room for 1 h before testing. All tests were performed at 4 time points (1, 3, 6, and 9 months) after the injection of BoNT-A or vehicle, and drug-induced rotations were additionally scored 0.5 months after BoNT-A or vehicle, always according to an identical time schedule (Figure 6). In rats drug-induced rotation tests were carried out 1, 2, 3, and 9 months after BoNT (*n* = 22) or vehicle (*n* = 6) injection.

#### 5.4.1. Drug-Induced Rotation Tests (Apomorphine, Amphetamine)

Mice rotations were assessed using an automated rotometer system (Rotometer UGO BASILE 43000; Ugo Basile Sri, Gemonio, Varese, Italy). Apomorphine was injected subcutaneously at a dosage of 0.5 mg/kg [123,124,125] (Teclapharm, Lüneburg, Germany) dissolved in 0.9% sterile saline. Three days later, D-amphetamine sulphate (Sigma Aldrich, München, Germany) was injected intraperitoneally at a dosage of 2.5 mg/kg [124,126,127] (Figure 6). Recording of apomorphine- and amphetamine-induced rotations began 5 min after drug injection and lasted 40 min. Rotations were defined as complete 360° turns and registered as net difference between the two directions per minute [128]. Anti-clockwise rotations were expressed by positive values, rotations in clockwise direction by negative values. Respective rotations in rats were induced by either 0.25 mg/kg apomorphine applied subcutaneously (Teclapharm, Germany) or 2.5 mg d-amphetamine sulfate (intraperitoneal; Sigma Aldrich, München, Germany), both solved in saline. Rotations were measured over 40 min after apomorphine and over 60 min after amphetamine in a self-constructed rotometer according to Ungerstedt and Arbuthnott [128].

#### 5.4.2. Spontaneous Motor Tests

Mice were tested in the cylinder test, stepping test, hindlimb clasping, and corridor task at 1, 3, 6, and 9 months after injection of BoNT-A or vehicle following a constant time table (Figure 6).

##### Cylinder Test

Forelimb preference was evaluated with the cylinder test as previously described [58,129]. Mice were placed in a glass cylinder (diameter 19 cm, height 20 cm) with mirrors placed behind to allow for a 360° view of every contact with the side of the cylinder. Sessions were taped with a video camera system (JVC, GZ-MG255E, Yokohama, Japan), and scored later. For each animal thirty consecutive forepaw contacts with the glass cylinder were evaluated by counting the initial contacts of the right or left paw. Then the ratio of left and right forepaw use was calculated. Evaluation of the videotapes was performed by an observer blinded to the animals’ identities.

##### Corridor Task

Lateralized sensorimotor integration and neglect were examined using the corridor task [61]. For our study we used a custom-made 60 cm long, 4 cm wide, and 15 cm high alleyway equipped with 10 pairs of adjacent pots with a diameter of 1 cm, placed at 5 cm intervals and containing 5 sugar pellets (Ain-76A Rodent Tablet 20 mg TestDiet, Richmond, IN, USA). Prior to testing, the mice were food-restricted for three days and maintained at 90% of free-feeding bodyweight during habituation and testing [130]. Animals were adapted to the apparatus for 10 min each on two consecutive days with some scattered sugar pellets along the floor of the corridor and started from different ends of the corridor each day. On the test day, mice were at first positioned in an identical, but empty corridor for 5 min for adaptation and then placed at the end of the testing corridor with bowls containing the pellet. Animals were allowed to move freely along the apparatus for 5 min to retrieve pellets placed on either side of their body. The number of ipsilateral (right side) and contralateral (left side) retrievals made by each mouse was calculated and the data were expressed as a percentage of left and right retrievals of the total number of retrievals. A “retrieval” was defined as a nose poke into a bowl, whether or not pellets were taken, and a new retrieval was counted by investigating a new pot [59,61].

##### Stepping Test

Forelimb akinesia was assessed according to Blume et al. [64] and modified by Heuer et al. [65] using an open table (1.5 m in length). Mice were tested three times in one day during the day light cycle. Each trial was recorded on video. As a first step for habituation to the test, mice were allowed to settle at one end of the table for 1–2 s, with all limbs on the table. Secondly, the experimenter gently lifted up the hindlimbs by pulling up on the tail leaving only the forepaws touching the table surface. Then, at a steady pace of 1 m in 3–4 s the experimenter pulled the animal the total test distance of 1 m backwards by the tail. Finally, the numbers of adjusting steps made with the left and right forepaws were counted offline in the videos.

##### Hindlimb Clasping

Hindlimb clasping is a marker of disease progression in a number of mouse models of neurodegeneration, including certain cerebellar ataxias [131] and parkinsonian mouse models [73,74], and was performed as previously described [72,73]. Outside the cage, each mouse was slowly lifted by the tail for 10 s and then lowered back to the surface. The hindlimbs were observed and the position of the hindlimbs was scored for each trial [72]. A score of 0 indicated that the hindlimbs were consistently splayed outwards and away from the abdomen. A score of 1 indicated that one hindlimb was retracted inwards towards the abdomen more than 50% of the trial period, a score of 2 indicated that both hindlimbs were partially retracted inwards towards the abdomen for at least 50% of the observation period. Mice were tested three times on one day and each trial was recorded on video.

### 5.5. Receptor Autoradiography and Histology

The autoradiographic procedure was performed according to standard protocols already published [132,133,134,135,136]. Cryosections of the respective brains were stained with cresyl-violet acetate (SIGMA C1791-5G) to verify the injection sites.

### 5.6. Statistical Analysis

Data of all behavioral tests were subjected to two-way ANOVA with repeated measurements. The Holm–Sidak test was used for post-hoc comparisons. The level of significance was set at *p* ≤ 0.05 for all statistical analyses. All statistical tests were done using SigmaPlot 11 Software (Systat Software, Inc., San Jose, CA 95110, USA).

## Supplementary Materials

The following are available online at http://www.mdpi.com/2072-6651/10/7/295/s1, Figure S1: Multi-receptor fingerprints of the caudate-putamen of mouse (blue) and rat (red), Figure S2: (A) Differential adjacency matrix of the rat and mouse basal ganglia. (B) Differential reciprocity matrix of rat and mouse basal ganglia.

## Abbreviations

6-OHDA	6-hydroxydopamine
BoNT-A	botulinum neurotoxin-A
ChAT	choline acetyltransferase
CPu	caudate-putamen
D_1_	dopamine D_1_ receptor
D_2_	dopamine D_2_ receptor
GABA	α-amino butyric acid
hemi-PD	hemiparkinsonian
ir	immunoreactive
nic	acetylcholine nicotinic α_4_β_2_ receptor
PD	Parkinson’s disease
SV2C	synaptic vesicle glycoprotein C
TH	tyrosine hydroxylase
