# Supplementary material for: Unilateral Botulinum Neurotoxin-A Injection into the Striatum of C57BL/6 Mice Leads to a Different Motor Behavior Compared with Rats
Source: Toxins (Basel). 2018 Jul 17;10(7):295. doi: 10.3390/toxins10070295 (PMC6070800; doi:10.3390/toxins10070295)
Supplement: Supplementary file 1 [file toxins-10-00295-s001.pdf]

Supplementary Materials: Unilateral Botulinum Neurotoxin-A Injection into the Striatum of C57BL/6 Mice Leads to a Different Motor Behavior Compared with Rats

Veronica Antipova, Andreas Wree, Carsten Holzmann, Teresa Mann, Nicola Palomero-Gallagher, Karl Zilles, Oliver Schmitt and Alexander Hawlitschka

Receptors and connectomics of the CPu of mouse and rat

Interspecies differences of transmitter systems in the striatum of naïve rats and mice were evaluated by multi-receptor fingerprints based on the densities of 18 transmitter binding sites (Figure S1) in the CPu of the Wistar rat (*n* = 6, data from [1–3]) and C57Bl/6 mouse (*n* = 6; data from Cremer et al. [4]). The fingerprints showed differences in the densities of several receptor types. However, we will here only discuss the receptors for neurotransmitters which have been identified as being essential for amphetamine-induced motor behavior (i.e., dopamine [5,6] and glutamate [7,8,9]). Thus, we were particularly interested in the species-specific differences in the densities (mean ± SD; fmol/mg protein) of D_1 (mouse: 4227 ± 537; rat: 2138 ± 137), D_2 (mouse: 2514 ± 212; rat: 1589 ± 70), mGluR2/3 (mouse: 8956 ± 2600; rat: 3472 ± 500), and AMPA (mouse: 590 ± 175; rat: 2390 ± 423) receptors. Interestingly, D_1, D_2 and mGluR2/3 densities were higher in the mouse than in the rat CPu, whereas the opposite holds true for the AMPA receptor. Taking these interspecies differences in receptor densities into account argues for considerable interspecies differences of transmitter systems in CPu between rats and mice.

Finally, the connectome of the mouse basal ganglia (MBG) generated in a high throughput tract tracing study published as Alan Atlas connectome [10] is compared to rat data. The connectivity data of the rat basal ganglia (RBG) has been collated within a metastudy of peer reviewed tract tracing publications [11]. Both connectomes were matched in *neuroVIISAS* to allow for pair wise comparisons ([12], https://www.ncbi.nlm.nih.gov/pubmed/?term=neuroVIISAS). The partial basal ganglia connectome of the rat contains bilaterally entire substantia nigra pars compacta and substantia nigra pars reticularis, ventrolateral thalamic nucleus, lateral globus pallidus, medial globus pallidus, striatum, subthalamic nucleus, lateral agranular frontal cortex, and medial agranular frontal cortex, i.e. the same regions as selected in the Alan Atlas connectome. The 18 basal ganglia regions of the RBG are interconnected by 169 connections, whereas the respective regions of the MBG are interconnected by 146 connections (Figure. S2A). The differential adjacency matrix reveals connections that occur only in mice (*n* = 42, marked in red), connections which occur only in rats (*n* = 71, marked in green), and those which occur in both species (*n* = 114, indicated by a white triangle). In one hemisphere 21 connections in mice differ from those of rats (Figure. S2A). The differential global network analysis indicates large similarities between rats and mice with regard to basic parameters such as average path length (MBG: 1.61, RBG: 1.45), average cluster coefficient (MGB: 0.52, RBG: 0.61) and small-worldness (MBG: 1.04, RBG: 1,11). However, the number of reciprocal connections in the RBG is 66 (reciprocity: 0.7052), and in the MBG it is 31 (reciprocity: 0.3068). The average flow coefficient in the MBG is 0.3411 and in the RBG 0.6571. In addition, the binary reciprocity matrices were computed using differential analysis (Figure. S2B). The matrix shows major differences (green elements) of reciprocal connections which occur exclusively in the rat connectome. Those reciprocal connections which occur exclusively in the mouse are indicated by red elements. Consensus reciprocities are indicated by white triangles. Thus, the connectomic data also clearly indicate an interspecific difference of the basal ganglia connectional architecture between both species, which may underlie the here reported differential behavioral outcome following intrastriatal BoNT-A injection.

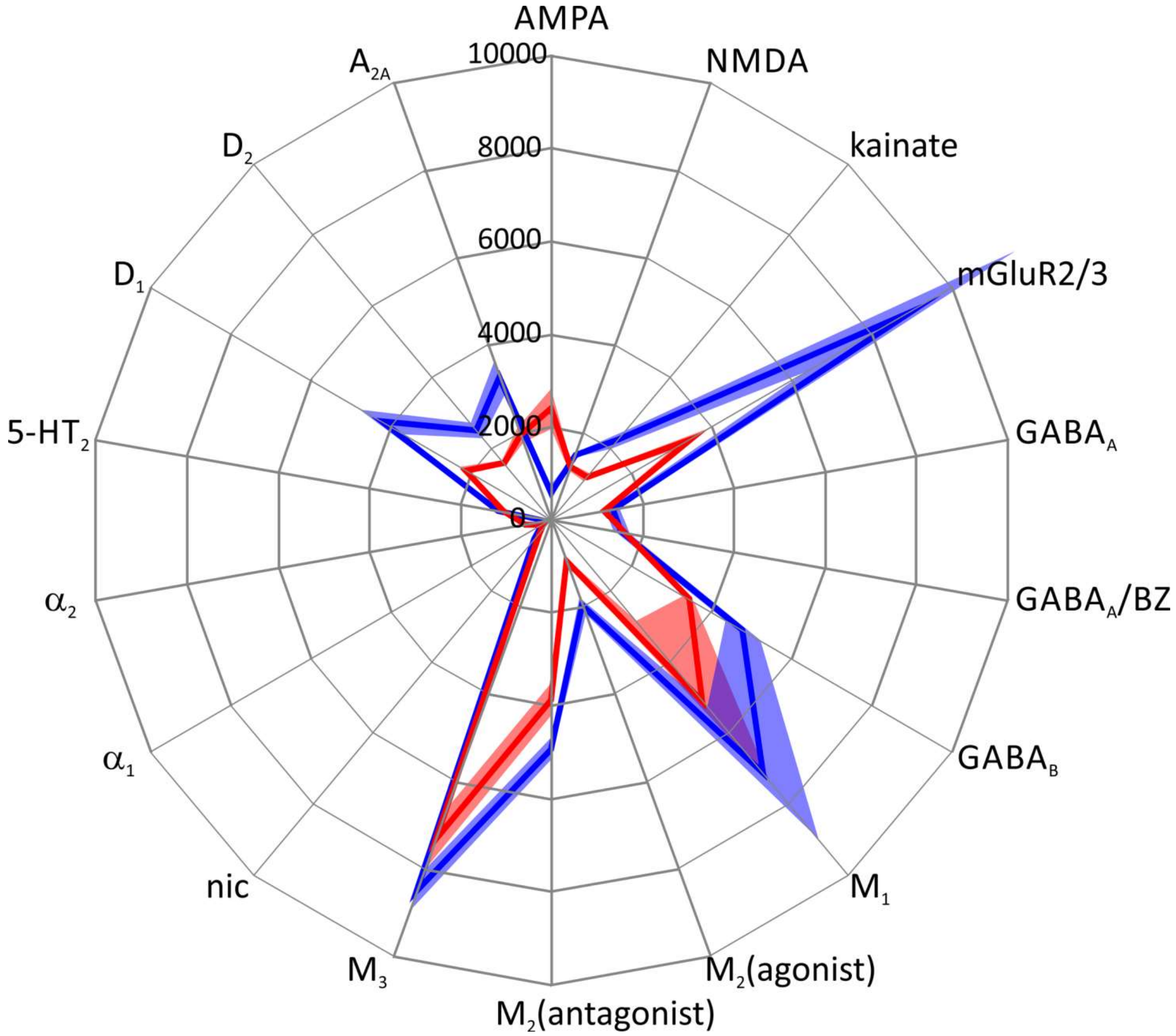

Figure S1. Multi-receptor fingerprints of the caudate-putamen of mouse (blue) and rat (red). Mean receptor densities (fmol/mg protein) are displayed in a polar coordinate plot. The lines connecting the mean densities create the shape of the fingerprints based on 18 different binding sites. AMPA: AMPA receptor labeled with [^{3}H]AMPA; NMDA: NMDA (N-methyl-D-aspartate) receptor labeled with [^{3}H]MK 801; kainate: kainate receptor labeled with [^{3}H]kainate; mGluR2/3: mGluR2/3 receptor labeled with [^{3}H]LY 341495; $GABA_A$: $GABA_A$ receptor labeled with [^{3}H]muscimol; $GABA_A$/BZ: $GABA_A$/BZ binding sites labeled with [^{3}H]flumazenil; $GABA_B$: $GABA_B$ receptor labeled with [^{3}H]CGP 54626; M_1: M_1 receptor labeled with [^{3}H]pirenzepine; M_2: M_2 receptor (agonistic binding site labeled with [^{3}H]oxotremorine-M; antagonistic binding site labeled with [^{3}H]AF-DX 384); M_3: M_3 receptor labeled with [^{3}H]4-DAMP; nic: $\alpha_4\beta_2$ nicotinic receptor labeled with [^{3}H]epibatidine; α_1: α_1 adrenoceptor labeled with [^{3}H]prazosin; α_2: α_2 adrenoceptor labeled with [^{3}H]UK 14,304; 5-HT_2: 5-HT_2 receptor labeled with [^{3}H]ketanserin; D_1: D_1 receptor labeled with [^{3}H]SCH 23390; D_2: D_2 receptor labeled with [^{3}H]raclopride; A_{2A}: A_{2A} receptor labeled with [^{3}H]ZM 241385. Shaded areas represent ± SD. Mouse data from Cremer et al. [4], rat values are from our own work [1–3].

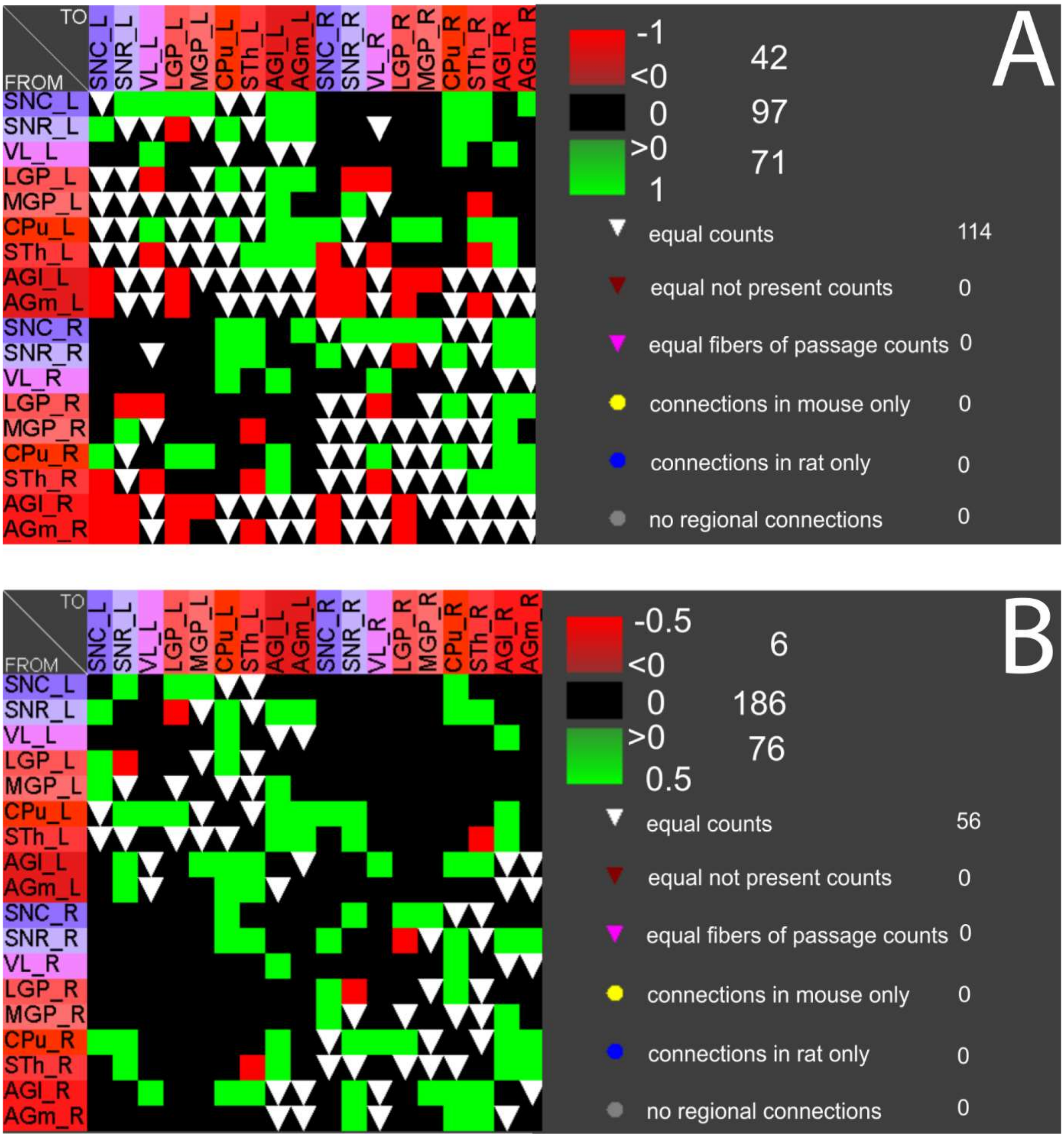

Figure S2. **(A)** Differential adjacency matrix of the rat and mouse basal ganglia. **(B)** Differential reciprocity matrix of rat and mouse basal ganglia. Abbreviations: AGl, lateral agranular prefrontal cortex; AGm, medial agranular prefrontal cortex; CPu, caudate-putamen; LGP, lateral globus pallidus; L, left hemisphere; MGP, medial globus pallidus; R, right hemisphere; SNC, substantia nigra pars compacta; SNR, substantia nigra part reticularis; VL, ventrolateral thalamic nucleus. Mouse data are from Oh et al. [10], rat connectome is from Schmitt et al. [11].

References

1. Mann, T.; Zilles, K.; Dikow, H.; Hellfritsch, A.; Cremer, M.; Piel, M.; Rösch, F.; Hawlitschka, A.; Schmitt, O.; Wree, A. Dopamine, Noradrenaline and Serotonin Receptor Densities in the Striatum of Hemiparkinsonian Rats following Botulinum Neurotoxin-A Injection. *Neuroscience* **2018**, *374*, 187–204, doi:10.1016/j.neuroscience.2018.01.053.
2. Mann, T.; Zilles, K.; Klawitter, F.; Cremer, M.; Hawlitschka, A.; Palomero-Gallagher, N.; Schmitt, O.; Wree, A. Acetylcholine neurotransmitter receptor densities in the striatum of hemiparkinsonian rats following Botulinum neurotoxin-A injection. *Front. Neuroanat.* in revision.
3. Mann, T.; Zilles, K.; Frederike, V.; Höhmann, K.; Hellfritsch, A.; Van Bonn, S.; Cremer, M.; Schmitt, O.;

Hawlitschka, A.; Wree, A. Glutamate, GABA and adenosine neurotransmitter receptor densities in the striatum of hemiparkinsonian rats following Botulinum neurotoxin-A injection. in preparation.

4. Cremer, J. N.; Amunts, K.; Graw, J.; Piel, M.; Rösch, F.; Zilles, K. Neurotransmitter receptor density changes in Pitx3ak mice - A model relevant to parkinson's disease. *Neuroscience* **2015**, *285*, 11–23, doi:10.1016/j.neuroscience.2014.10.050.
5. Gershanik, O.; Heikkila, R. E.; Duvoisin, R. C. Behavioral correlations of dopamine receptor activation. *Neurology* **1983**, *33*, 1489–1492, doi:10.1212/WNL.33.11.1489.
6. Carlson, J. H.; Bergstrom, D. A.; Walters, J. R. Stimulation of both D1 and D2 dopamine receptors appears necessary for full expression of postsynaptic effects of dopamine agonists: a neurophysiological study. *Brain Res.* **1987**, *400*, 205–218, doi:10.1016/0006-8993(87)90619-6.
7. Yu, M.-F.; Chien, C.-L.; Lee, W.-T.; Yin, H.-S. Effects of acute amphetamine administration on AMPA-mediated synaptic activity and expression of AMPA receptor subunit 2 of brain neurons. *J. Mol. Neurosci.* **2005**, *25*, 171–181, doi:10.1385/JMN:25:2:171.
8. Miele, M.; Mura, M. A.; Enrico, P.; Esposito, G.; Serra, P. A.; Migheli, R.; Zangani, D.; Miele, E.; Desole, M. S. On the mechanism of d-amphetamine-induced changes in glutamate, ascorbic acid and uric acid release in the striatum of freely moving rats. *Br. J. Pharmacol.* **2000**, *129*, 582–588, doi:10.1038/sj.bjp.0703066.
9. Vanover, K. E. Effects of AMPA receptor antagonists on dopamine-mediated behaviors in mice. *Psychopharmacology (Berl).* **1998**, *136*, 123–131, doi:10.1007/s002130050547.
10. Oh, S. W.; Harris, J. A.; Ng, L.; Winslow, B.; Cain, N.; Mihalas, S.; Wang, Q.; Lau, C.; Kuan, L.; Henry, A. M.; Mortrud, M. T.; Ouellette, B.; Nguyen, T. N.; Sorensen, S. A.; Slaughterbeck, C. R.; Wakeman, W.; Li, Y.; Feng, D.; Ho, A.; Nicholas, E.; Hirokawa, K. E.; Bohn, P.; Joines, K. M.; Peng, H.; Hawrylycz, M. J.; Phillips, J. W.; Hohmann, J. G.; Wohnoutka, P.; Gerfen, C. R.; Koch, C.; Bernard, A.; Dang, C.; Jones, A. R.; Zeng, H. A mesoscale connectome of the mouse brain. *Nature* **2014**, *508*, 207–214, doi:10.1038/nature13186.
11. Schmitt, O.; Eipert, P.; Kettlitz, R.; Leßmann, F.; Wree, A. The connectome of the basal ganglia. *Brain Struct. Funct.* **2016**, *221*, 753–814, doi:10.1007/s00429-014-0936-0.
12. Schmitt, O.; Eipert, P. NeuroVIISAS: Approaching multiscale simulation of the rat connectome. *Neuroinformatics* **2012**, *10*, 243–267, doi:10.1007/s12021-012-9141-6.
